# Synthesis of Lignin and PLA/PBAT Films: Biodegradability and Environmental Impacts

**DOI:** 10.3390/polym18070793

**Published:** 2026-03-25

**Authors:** Nutchapon Chiarasamran, Ronnachai Jitsamut, Paweena Prapainainar, Anusith Thanapimmetha, Maythee Saisriyoot, Suraini Abd-Aziz, Chanin Khomlaem, Beom Soo Kim, Penjit Srinophakun

**Affiliations:** 1 Department of Chemical Engineering, Faculty of Engineering, Kasetsart University, Bangkok 10900, Thailand; fengnpc@ku.ac.th (N.C.); jam1._ronnachai@hotmail.com (R.J.); fengpwn@ku.ac.th (P.P.); fengjrc@gmail.com (A.T.); fengmts@ku.ac.th (M.S.); 2 Department of Bioprocess Technology, Faculty of Biotechnology and Biomolecular Sciences, Universiti Putra Malaysia, Serdang 43400, Selangor, Malaysia; suraini@upm.edu.my; 3 Sustainable Materials Laboratory, Department of Materials Science and Engineering, Faculty of Engineering and Industrial Technology, Silpakorn University, Sanamchandra Palace Campus, Nakhon Pathom 73000, Thailand; nament99@gmail.com; 4 Department of Chemical Engineering, Chungbuk National University, Cheongju 28644, Chungbuk, Republic of Korea

**Keywords:** PLA, PBAT, lignin, biodegradation, compatibilizer, life cycle assessment

## Abstract

We investigated the synthesis and characterization of biodegradable films composed of poly (lactic acid) (PLA) and poly(butylene adipate-co-terephthalate) (PBAT), with lignin as a natural additive and dicumyl peroxide (DCP) as a compatibilizer. The PLA/PBAT ratio of 70:30 was optimized and the DCP was incorporated at different concentrations to enhance interfacial adhesion. The effects of lignin addition (0.005–0.02%) on the mechanical, thermal, and biodegradation properties were evaluated using SEM, FTIR, XRD, and TGA analyses. The optimal formulation had improved tensile strength, elongation at break, and thermal stability, with the highest degradation rate of 44.22% after 90 days of soil burial. Life cycle assessment using SimaPro software (SimaPro 9.1.1.1) and ReCiPe 2016 Midpoint indicated that the film containing 0.005% lignin had the lowest environmental impact. The primary environmental concerns were marine and freshwater ecotoxicity, associated with solvent use. Based on the results, incorporating small amounts of lignin enhanced the biodegradability and reduced the environmental footprint of the PLA/PBAT films, highlighting their potential for sustainable packaging applications.

## 1. Introduction

In recent decades, the plastics and associated products industries have experienced exponential growth, driven by population growth and rising demand across consumer and industrial sectors [1,2]. Petroleum-based plastics, such as polyethylene, polyvinyl chloride, and polypropylene, are used widely in industry because of their toughness, flexibility, transparency, and thermal stability, while their low cost makes them popular for diverse everyday applications [3]. Plastic films can be produced from a group of widely used polyolefins; however, the increased use of these plastics has had a severe negative impact on the environment and ecosystems, as well as harmful effects on living organisms and marine animals. Therefore, alternatives to traditional petroleum-based plastics have been sought, specifically biodegradable bioplastics [4]. One polymer of interest is poly(lactic acid) (PLA), a semi-crystalline polymer that is non-toxic and biodegradable. It is produced from lactic acid, which can be synthesized chemically or via fermentation processes primarily involving sugars or starches such as those derived from sugarcane, corn, wheat, and biomass waste [5].

However, due to PLA’s high brittleness and limited impact resistance, studies have explored blending it with other polymers. For example, by adding a flexible polymer, such as poly(butylene adipate-co-terephthalate) (PBAT), which is strong, easy to process, and highly adaptable, the blend will better withstand stress than PLA alone [6]. According to the research, because the two polymers are not compatible, a compatibilizer must be selected to aid the blending process. Dicumyl peroxide (DCP) has been used as a compatibilization aid in the PLA/PBAT system. During the solvent evaporation stage and subsequent mild thermal treatment, partial decomposition of the DCP may occur, generating reactive species that promote interfacial interactions between the PLA and PBAT phases. These interactions can improve compatibility and interfacial adhesion within the polymer blend [7].

Lignin is a complex biopolymer with a high molecular weight (1000–>100,000 g/mol) that is composed of carbon, hydrogen, and oxygen that form various subunits. It is obtained from biomass materials and is the second most abundant biopolymer in nature after cellulose [8]. Lignin is a naturally occurring aromatic biopolymer that can be degraded by specific microorganisms under favorable environmental conditions, although its degradation rate is generally slower due to its complex structure [3]. Lignin, being a phenolic compound, is insoluble in water. Naturally, lignin is colorless or pale yellow; however, when treated with acids or bases, its color changes to dark brown. The basic building blocks of lignin are monolignols, with the main types being p-coumaroyl alcohol, coniferyl alcohol, and sinapyl alcohol [9]. Lignin contains various functional groups, such as phenolic hydroxyl (-OH), aliphatic hydroxyl (-OH), methoxy (-OCH_3_), and carbonyl groups, that can interact with the ester groups in PLA and PBAT through hydrogen bonding and interfacial interactions, thereby improving compatibility between the polymer phases. These compounds exhibit antimicrobial activity, inhibiting the growth of certain microorganisms, as well as antioxidant activity to resist free radicals. Additionally, lignin acts as an ultraviolet (UV) absorber and a reinforcing agent [10].

The current research focused on developing a hybridization film made from lignin, PLA, and PBAT to improve the composite’s physical and mechanical properties. Due to the incompatibility of PLA and PBAT, DCP was used at varying concentrations to improve intermolecular adhesion between the PLA and PBAT polymers. Tetrahydrofuran (THF) was used to mix the PLA, PBAT, and DCP, and dimethyl sulfoxide (DMSO) was used to dissolve the lignin. The effects on mechanical properties and biodegradability were investigated at various lignin loadings. Finally, a life cycle assessment (LCA) was performed to evaluate the environmental impacts of the lignin PLA/PBAT film. In addition, the potential of making the film more environmentally friendly was examined by assessing the impacts of the chemicals used in the process.

## 2. Materials and Methods

Poly(lactic acid) (PLA), grade LUMINY LX175, with a melt flow index of 8 g per 10 min, was sourced from Total Corbion PLA Co, Ltd. (Rayong, Thailand). Poly(butylene adipate-co-terephthalate) (PBAT), grade Ecoflex F Blend C1200, with a melt flow index of 3.8 g per 10 min, was supplied by BASF (Bangkok, Thailand). Dealkaline lignin, grade L0045, and dimethyl sulfoxide (DMSO), 99.5% pure, were provided by TS Interlab Part., Ltd. (Bangkok, Thailand). Dicumyl peroxide (DCP), 98% pure, with a molecular weight of 270.37 and a density of 1.56, was purchased from Lab Systems Ltd. (Bangkok, Thailand). Tetrahydrofuran (THF), 99.5% pure, analytical grade, with a molecular weight of 72.11 g/mol, was also supplied by Lab Systems Co., Ltd. (Bangkok, Thailand). 

The life cycle assessment (LCA) was performed using SimaPro software (SimaPro 9.1.1.1, PRé Sustainability, Amersfoort, The Netherlands) with the ReCiPe 2016 Midpoint method, following standard LCA methodology reported in the literature [5].

### 2.1. Preparation of Polymer Films

The sample codes of the PLA/PBAT polymer blend with added DCP at concentrations of 0, 0.01, 0.02, and 0.03 wt.% are shown in Table 1. The PLA and PBAT pellets were dried at 60 °C for 6 h. At the highest possible ratio of PLA/PBAT, the dried PLA and PBAT pellet at 70:30 was fixed in the study. A total weight of 3 g was used to prepare PLA/PBAT/DCP via solution casting under continuous heating using a magnetic stirrer to obtain a homogeneous blend for 60 min. Subsequently, DCP was added to the PLA/PBAT solution and further stirred for 30 min. The polymer blend solution was poured onto a glass plate and placed on a stirrer with slight heating to aid solvent evaporation. Finally, the mixture was placed in an oven at 90 °C to remove any residual solvent.

**Figure 1:**
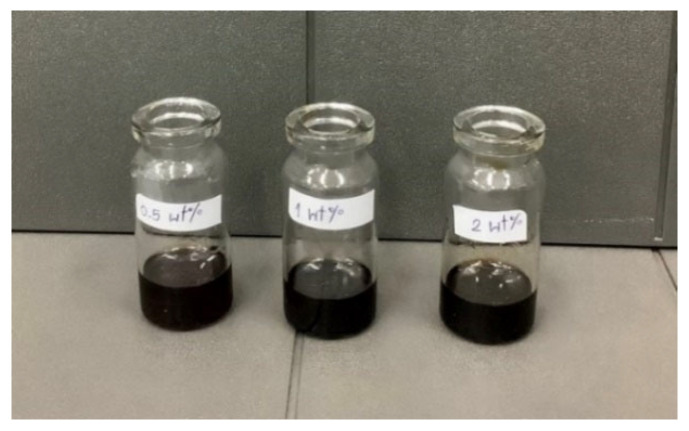
Dark brown color of dissolved lignin in DMSO at lignin concentrations of 0.005, 0.01, and 0.02% (from left to right, respectively).

For the lignin solution blending process, the PLA and PBAT pellets were blended in a 70:30 ratio with 0.03% DCP and mixed using the solution-casting method with tetrahydrofuran (THF) as the solvent. After stirring for 90 min, the lignin in DMSO was added at concentrations of 0.005, 0.01, and 0.02% (by weight), each measuring 3 g, as specified in Table 2, and the mixture was stirred until homogeneous. The prepared polymer solutions were deep brown in color (Figure 1). Then, the mixtures were placed in an oven at 90 °C to evaporate any remaining solvent from the film.

### 2.2. Characterization

#### 2.2.1. Mechanical Properties

Mechanical properties were evaluated using a universal testing machine, with tests conducted in accordance with ASTM D638 standards [11]. Briefly, the sample size was 1 × 6 cm (length by width), and the film thickness was measured at three positions, each measured three times to calculate the average thickness. For each ratio, at least five specimens were used. Testing was performed with a 40 mm grip distance, a stretching rate of 10 mm/min, and a pulling force of 0.9 kN. The machine reported values for modulus, tensile strength, and elongation at break.

#### 2.2.2. Fourier Transform Infrared (FTIR) Spectroscopy

Chemical structures and organic and inorganic materials, as well as contaminants, were determined by FTIR (Bruker, Billerica, MA, USA). The FTIR spectra were recorded in the 500–4000 cm^−1^ wave number range.

#### 2.2.3. Morphological Study

Morphological characteristics were examined using scanning electron microscopy (SEM) on cross-sections of the samples. First, each sample was frozen using liquid nitrogen and then fractured to observe its morphology based on its cross-sectional area. In addition, portions of the sample were mounted onto stubs to study the surface morphology after accelerated weathering. Surface samples and fracture surfaces were coated with a thin layer of gold to enhance conductivity before testing [12].

#### 2.2.4. Thermogravimetric (TGA) Analysis

The thermal stability was studied using a Thermogravimetric Analyzer (TGA/DSC 3+ LF STAR system, Mettler Toledo, Greifensee, Switzerland) under a nitrogen atmosphere. The test was conducted over a temperature range of 25–600 °C at a heating rate of 10 °C per minute. The TGA data included the maximum degradation temperature (T_d_), which indicates the temperature at which the material undergoes the highest rate of thermal decomposition [13].

#### 2.2.5. X-Ray Diffraction (XRD) Analysis

The crystallinity of the prepared polymer films containing dicumyl peroxide and lignin was studied using XRD. Each 3 × 3 cm (width by length) polymer film sample was prepared and placed in the test tray. Testing began at an initial angle of 2ϴ = 3° and ended at 2ϴ = 50°, with a scan speed of 2 degrees per minute. The electric current was 40 A and the voltage was set to 45 kV. The results were presented as a graph showing the relationship between peak intensity and diffraction angle [14].

#### 2.2.6. Chemical Structure Analysis

The chemical structure of the polymer films was analyzed using a UV-vis spectrophotometer (HACH DR 6000, Loveland, CO, USA). Each sample was examined across a wavelength range of 900–200 nm in absorption mode [15]. The results were presented in a graph showing the relationship between absorbance and wavelength (nm). Additionally, the haze value was calculated using Equation (1).

(1) $$Opacity=\frac{Absorbance\;at\;600\;nm}{Film\;thickness}$$

#### 2.2.7. Biodegradation Test

The study of soil degradation involved cutting samples to 2 × 2 cm (width by length), testing them at a soil depth of 22 cm. Soil conditions were controlled through periodic watering and referred to the procedure of Balakrishnan et al. [16] and Sirivechphongkul et al. [17]. Samples were removed from the soil at intervals of 30, 60, and 90 days to analyze the percentage of weight loss after burial, calculated using Equation (2).

(2) $$Weight\;loss\left(\%\right)=\frac{Initial\;weight-Final\;weight}{Initial\;weight}\times 100$$

#### 2.2.8. Environmental Impacts

The functional unit of this study was 1 kg of film produced. There were 4 scenarios for 4 different types of films, namely PLA/PBAT, PLA/PBAT with lignin 0.5% (PLA/PBAT LN^0.5^), PLA/PBAT with lignin 1.0% (PLA/PBAT/LN^1^), and PLA/PBAT with lignin 2.0% (PLA/PBAT/LN^2^). All cases were analyzed gate-to-gate, with input and output quantities of raw materials and energy per unit of plastic resin production to film product, using SimaPro software (SimaPro 9.1.1.1, PRé Sustainability, Amersfoort, The Netherlands). The life cycle impact assessment procedure, based on the Ecoinvent database and the environmental impact assessment, used ReCiPe 2016 Midpoint/Endpoint (H) V1.04 software. All data were obtained from the database except for PBAT. Electrical use for polymer mixing and film making was reported by Sirivechphongkul et al. [17].

## 3. Results and Discussion

### 3.1. FTIR Analysis

**Figure 2:**
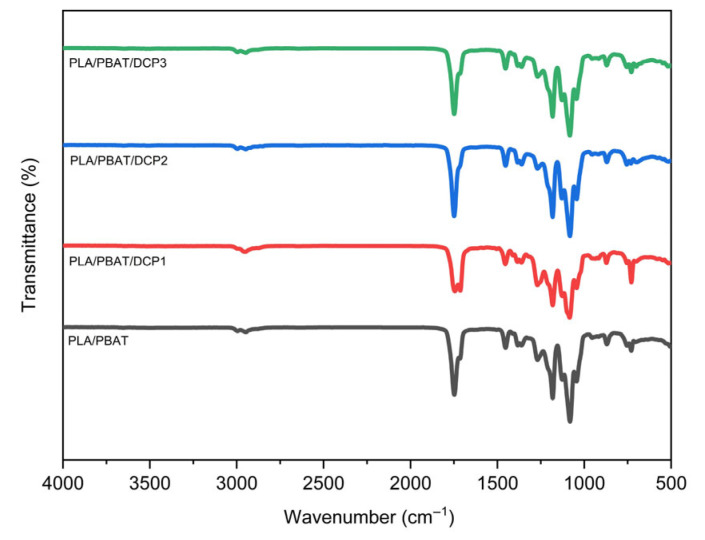
FTIR spectra of PLA/PBAT polymer system with varying DCP ratios, namely PLA/PBAT/DCP1 (0.01 wt.%), PLA/PBAT/DCP2 (0.02 wt.%), and PLA/PBAT/DCP3 (0.03 wt.%).

Analysis of the polymer film’s chemical structure, as shown in Figure 2, revealed characteristic peaks at wavenumbers 2996, 1747, 1455, and 1180 cm^−1^, corresponding to C-H stretching, C=O stretching, CH3 bending, and C-O stretching, respectively, which were consistent with other research findings [18]. When the DCP was added to the PLA/PBAT system, no changes in chemical structure were observed based on the FTIR analysis, consistent with other research findings [19].

**Figure 3:**
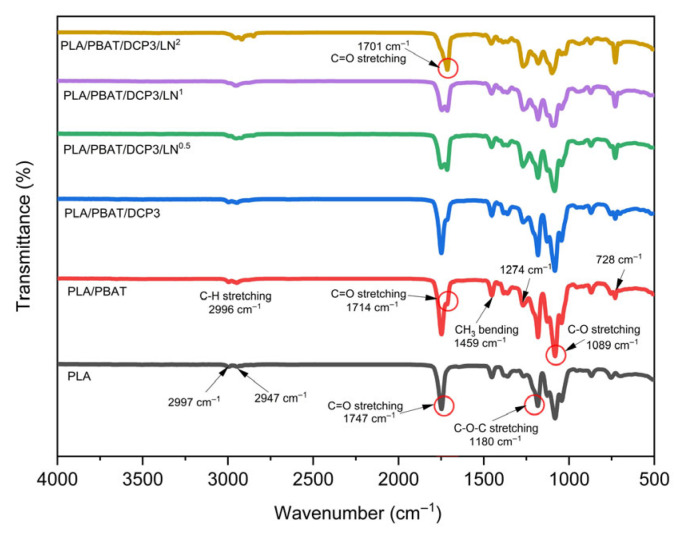
FTIR spectra of PLA, PLA/PBAT, PLA/PBAT/DCP3 (0.03 wt.% DCP), and various lignin concentrations, PLA/PBAT/DCP1/LN^0.5^ (0.005 wt.% lignin), PLA/PBAT/DCP1/LN^1^ (0.01 wt.% lignin), and PLA/PBAT/DCP1/LN^2^ (0.02 wt.% lignin).

When a polymer compatibility enhancer (such as DCP) was added to the PLA/PBAT film system at a concentration of 0.03%, the peak characteristics remained unchanged. However, after the lignin had been added to the polymer, as shown in Figure 3, there were slight shifts in the characteristic peaks after the lignin incorporation. The C=O stretching band shifted from approximately 1747 cm^−1^ to 1750 cm^−1^, while the C-O-C stretching band around 1150 cm^−1^ shifted slightly to higher wavenumbers, suggesting interactions between lignin functional groups and the polymer matrix. Specifically, the peaks at 1150 cm^−1^ (C-O-C stretching) and 1750 cm^−1^ (C=O stretching), along with the C-H aromatic ring peaks of the PBAT polymer, shift, indicating an interaction between the lignin and the polymer matrix.

### 3.2. Morphological Characterization

**Figure 4:**
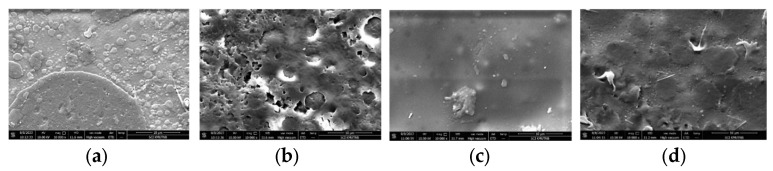
SEM images of PLA/PBAT polymer films with different DCP concentrations: (**a**) 0%; (**b**) 0.01%; (**c**) 0.02%; (**d**) 0.03%.

The morphology of the PLA/PBAT polymer film was analyzed using scanning electron microscopy (SEM). Upon the addition of dicumyl peroxide (DCP) at varying concentrations, as shown in Figure 4, the film’s morphology improved due to compatibility and adhesion between the PLA and PBAT phases. The enhanced interaction between these two polymers suggested that peroxide acted as a crosslinking agent, promoting better phase mixing and interfacial bonding. The addition of DCP acted as a free radical initiator, which reduced the particle size of PBAT and enhanced the adhesion between the polymer molecules of PLA and PBAT (70:30). Furthermore, the addition of the DCP in various concentrations (0.1–0.5%) resulted in stronger adhesion between the two polymers due to the melting and blending processes that enhanced the compatibility of the polymer blend [20,21].

**Figure 5:**
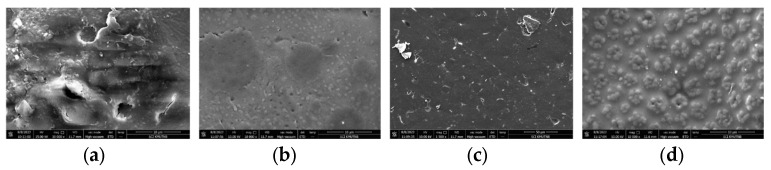
SEM images of PLA/PBAT/DCP polymer films with varying lignin concentrations: (**a**) 0%; (**b**) 0.005%; (**c**) 0.01%; (**d**) 0.02%.

We examined the cross-sectional images of the PLA/PBAT polymer blend with the addition of the DCP and varying amounts of lignin. As shown in Figure 5, there was a notable change in morphology. The lignin particle size increased after adding lignin at concentrations of 0.01–0.02%, leading to the formation of bonds between the lignin molecules. The formation of these bonds reduced the surface area available for force transfer between the main polymer matrix and the lignin particles. Furthermore, the reaction between lignin molecules made the composite more brittle, reducing the tensile strength of the blend. These results were consistent with another study indicating that lignin addition in polymer blends led to phase separation and decreased ductility, which negatively affected the overall mechanical properties such as toughness and elongation at break [4]. The current study involved the incorporation of PLA into a PBAT/lignin matrix, which enhanced both the strength and flexibility of the polymer blend.

In this system, lignin acted as a crosslinker between the PLA and PBAT, improving their compatibility. Based on the experimental results, the lignin dissolved in methanol had good compatibility and dispersion within the polymer blend, leading to better interfacial bonding between the PLA and PBAT. This finding aligned with another study that highlighted the benefits of lignin as a plasticizer and compatibilizer for improving the mechanical properties of biodegradable polymer blends, as well as its ability to aid in uniform dispersion within the matrix [22].

### 3.3. XRD Analysis

**Figure 6:**
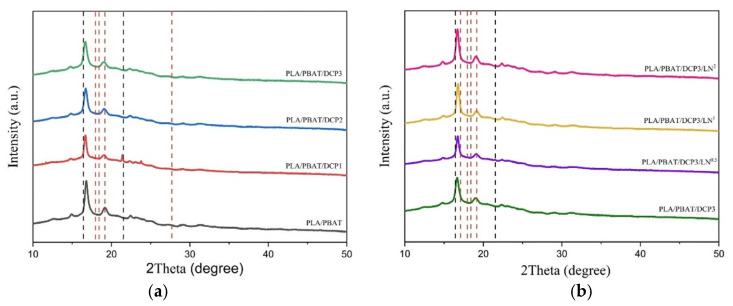
XRD patterns of PLA/PBAT polymer film: (**a**) with DCP amounts of 0, 0.01, 0.02, and 0.03% (black—PLA/PBAT; red—PLA/PBAT/DCP1; blue—PLAT/PBAT/DCP2; green—PLA/PBAT/DCP3); (**b**) with lignin amounts of 0, 0.005, 0.01, and 0.02% (green—PLA/PBAT/DCP3; purple—PLA/PBAT/DCP3/LN^0.5^; yellow—PLA/PBAT/DCP3/LN^1^; red—PLA/PBAT/DCP3/LN^2^).

The XRD patterns of the polymer blended films between PLA and PBAT, shown in Figure 6a, revealed the characteristic peaks of PLA at 2ϴ angles of 16.81° and 29.05° and of PBAT at 2ϴ angles of 17.72°, 19.90°, 21.04°, and 22.91°. Based on the study of the crystalline structure of the PLA and PBAT, there were characteristic peaks at 2ϴ angles of 16.7° for the PLA and 17.6°, 20.5°, 23.4°, and 25° for the PBAT. After adding the different amounts of DCP to the polymer film, the characteristic peak positions of the PLA shifted (Figure 6a). Specifically, at 0.01, 0.02 and 0.03% DCP, the peak positions shifted slightly to 16.68°, 16.74°, and 16.66°, respectively, indicating that the addition of the DCP did not greatly affect the crystalline structure of the PLA and PBAT (the peaks remained sharp and did not broaden). Adding lignin (0.005–0.02%) to the PLA and PBAT blends, as shown in Figure 6b, or the addition of the compatibilizer (DCP at 0.03%) produced a difference in the crystallization behavior of the polymer. The incorporation of lignin slightly modified the crystallization behavior of the polymer blend as the lignin particles acted as heterogeneous nucleating sites, facilitating the crystallization of PLA domains and slightly increasing the overall crystallinity of the composite [23]. The dashed lines in Figure 6 represent the characteristic diffraction peaks of PLA (black) and PBAT (red).

### 3.4. Mechanical Behavior

**Figure 7:**
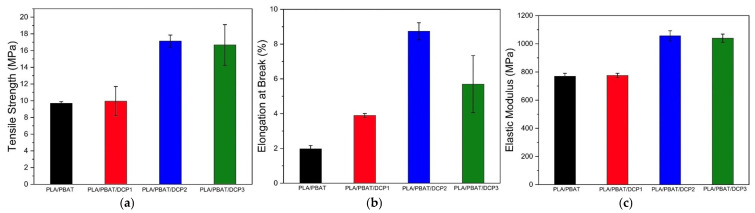
Mechanical properties of polymer films with varying DCP concentrations of 0, 0.01, 0.02, and 0.03%: (**a**) Tensile strength; (**b**) elongation at break; (**c**) elastic modulus. Error bars indicate ± standard error.

The mechanical properties were evaluated using tensile testing, as shown in Figure 7. The PLA/PBAT polymer blend film without DCP had tensile strength, elongation at break, and elastic modulus values of 9.69 MPa, 1.978%, and 769 MPa, respectively. Deng et al. [24] investigated the effect of PLA/PBAT blending ratios on crystallization behavior. They reported a higher degree of crystallization with increasing amounts of PBAT in the formulation. Hence, as shown in Figure 7, there were increases in the density, stiffness (modulus), and tensile strength of the materials. Adding DCP from 0.01 to 0.03% further enhanced mechanical properties, particularly the elongation at break of the composites, which increased by 3–4 times when the DCP was added at 0.02 and 0.03%. DCP, as a free-radical initiator during melt blending, triggered reactions that created a bridge between the PLA and PBAT. As a result, phase separation was reduced, and interfacial adhesion was improved, as indicated by the SEM results in Figure 5. With a higher DCP content, the fracture surface of the film became smoother, suggesting easier breakage. Similar results were reported elsewhere [20].

**Figure 8:**
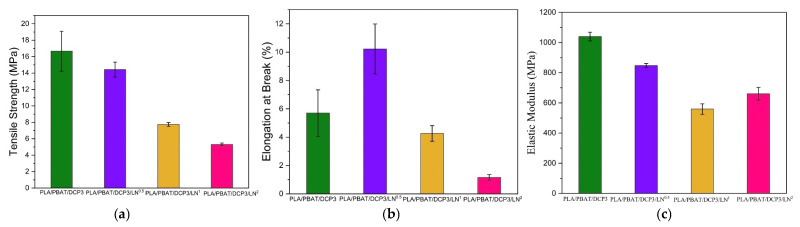
Mechanical properties of polymer films with DCP concentration of 0.03% and varying lignin concentrations of 0, 0.005, 0.01, and 0.02%: (**a**) tensile strength; (**b**) elongation at break; (**c**) elastic modulus.

In the current study, the lignin had no notable effect on reducing the tensile strength, elongation at break, or material flexibility; however, adding more lignin would further degrade these properties (Figure 8). Lignin improved the compatibility between the PLA and PBAT [25], since as a UV absorber, lignin provided ultraviolet shielding and enhanced water vapor barrier properties of the composite film. Lignin could serve as a green compatibilizer for PLA/PBAT composites, with its antimicrobial property making these biodegradable plastics ideal for outdoor applications and protective food packaging [26,27].

Based on the mechanical properties shown in Figure 7 and Figure 8, the synthesized PLA/PBAT/DCP3/LN^0.5^ could be applied in the high-density polyethylene (HDPE) category; specifically, the elastic modulus was within the range for HDPE applications. Low tensile strength can be improved by adding reinforcement fibers, such as graphene [21]. However, the elongation at break was quite low. Therefore, PLA/PBAT/DCP3/LN^0.5^ could be used in easy-tear plastic film or rigid packaging with antimicrobial properties. Furthermore, the brown color of the lignin would protect products from light-induced oxidation.

### 3.5. Thermal Analysis

**Figure 9:**
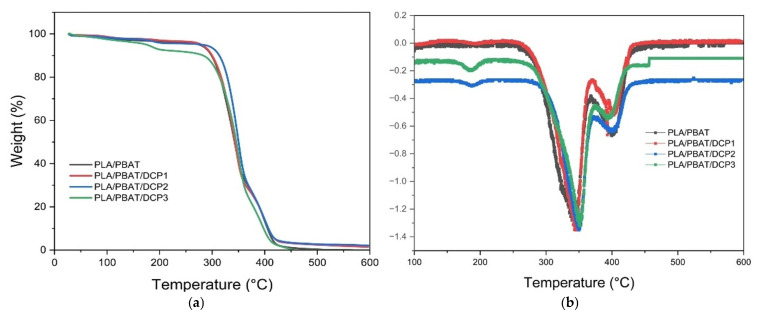
TGA thermograms of PLA/PBAT polymer films with different DCP concentrations (0, 0.01, 0.02, and 0.03%): (**a**) TGA; (**b**) DTG.

Thermal stability testing based on TGA, as shown in Figure 9 and Table 3, revealed that the PBAT had better thermal stability than the PLA, which could be due to the longer chain segments and aromatic structural groups of the former. In the PLA and PBAT blends, the addition of the DCP caused only a slight change in thermal stability. For only PLA and PBAT, the thermal decomposition temperatures were T_5%_ = 276.8, T_50%_ = 342.1, and T_d_ = 420.7 °C. Comparing the blends with and without DCP, the thermal decomposition temperature increased slightly due to improved interfacial bonding between the two phases, thereby enhancing overall material strength. However, at DCP 0.03%, T_5%_ was lower than for the other conditions, as the earlier mass loss in this sample might have been due to decomposition or overdosing of DCP.

**Figure 10:**
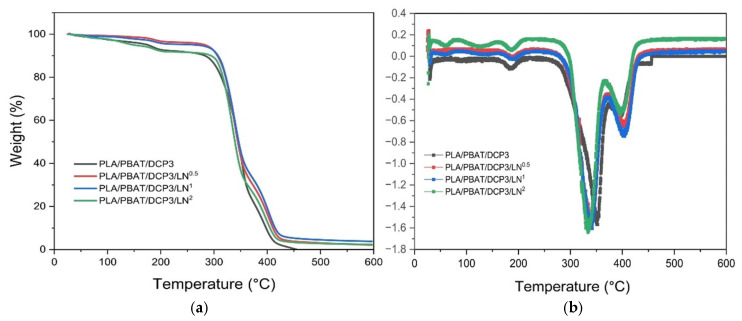
TGA thermograms of PLA/PBAT/DCP polymer films with different lignin concentrations (0.005, 0.01, and 0.02%): (**a**) TGA; (**b**) DTG.

After adding lignin to the PLA and PBAT blends at a DCP concentration of 0.03%, their thermal stability increased slightly, with variations in the lignin concentrations of 0.005, 0.01, and 0.02%, as shown in Figure 10 and Table 4. At a lignin concentration of 0.02%, T_5%_ was lower than at the other lignin concentrations, which might have been due to the decomposition of the excess lignin in the composites. Char residue confirmed the presence of lignin and DCP. Table 4 shows the relation between char residue and lignin concentrations. Increasing the lignin concentration from 0.05 to 0.1% resulted in more char residue, consistent with the results of another study where ferulic acid was added [27].

### 3.6. Film Color and Opacity

**Figure 11:**
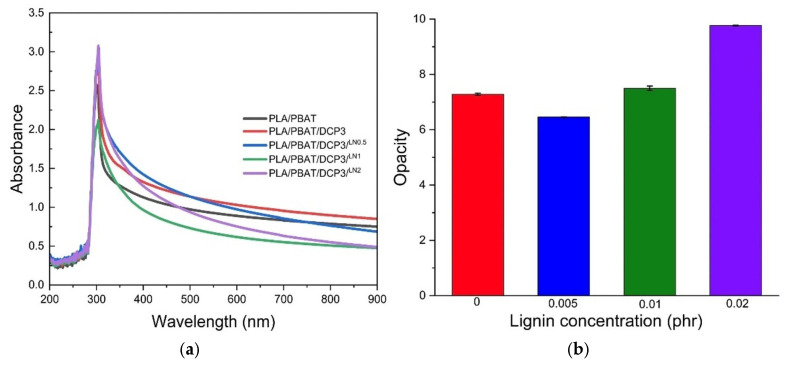
Light absorption (**a**) and opacity (**b**) of polymer film at various lignin concentrations.

**Figure 12:**
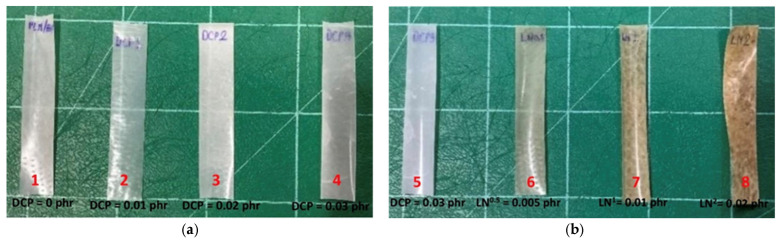
Opacity of the polymer film to visibility: (**a**) DCP at (**1**) 0%, (**2**) 0.01%, (**3**) 0.02%, and (**4**) 0.03%; (**b**) lignin amounts at (**5**) 0%, (**6**) 0.005%, (**7**) 0.01%, and (**8**) 0.02%.

The UV-vis absorption and opacity of the PLA/PBAT, PLA/PBAT/DCP, and PLA/PBAT/DCP/LN polymers were studied using a UV-vis spectrometer. All composites had low absorbance at 600 nm (Figure 11a), a low absorption edge threshold, and a band gap energy greater than 2 eV (Table 5), indicating that the materials would be insulators with low opacity (Figure 11b). Increasing the lignin content further enhanced the polymer film’s UV absorption at longer wavelengths, suggesting that lignin-enriched polymer films would be effective at absorbing more UV radiation [10,28]. This material would be a transparent insulator because its band gap energy was higher than 3.1 (Table 5). Higher levels of lignin addition increased the material’s opacity (Figure 12), corresponding well with the results from Figure 11b.

### 3.7. Soil Burial Degradation

**Figure 13:**
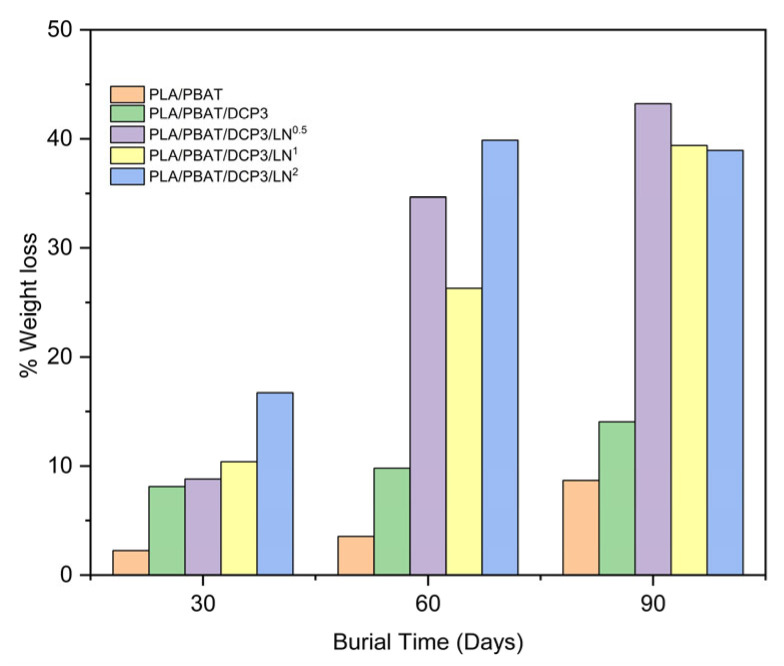
Biodegradation through soil burial based on weight change in the film (% weight loss) at 30, 60, and 90 days.

**Figure 14:**
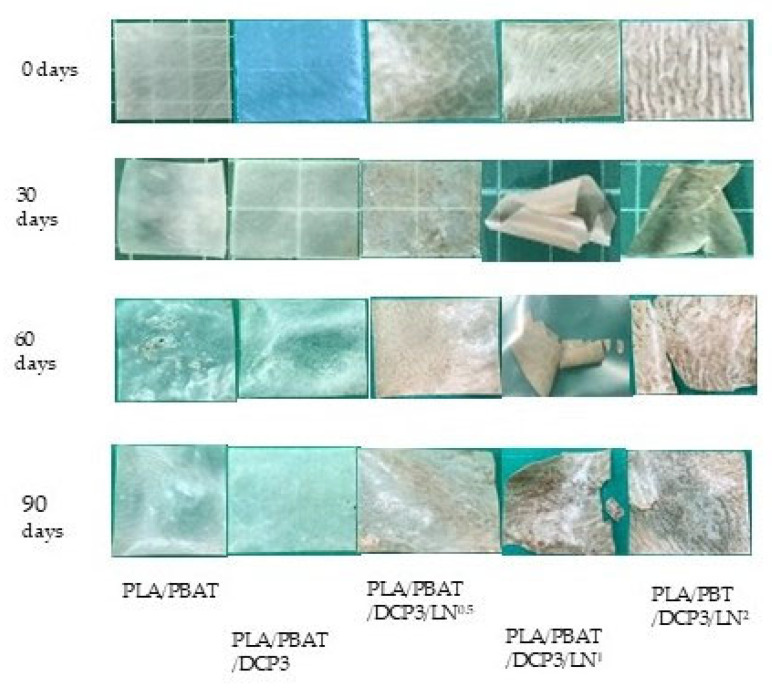
Polymer images of samples after soil burial for 30, 60, and 90 days.

The biodegradability of the buried samples of the composite polymers of PLA and PBAT was evaluated based on observations at 0, 30, 60, and 90 days. The weight loss during the burial period was recorded (Figure 13), and images were taken periodically (Figure 14). It was observed that adding lignin increased biodegradability. The lignin-containing polymer blends had degradation percentages of 8.8%, 10.4%, and 16.7%, respectively, at 30 days. From Figure 13, it was found that the percentage of weight loss increased after 90 days of burial following the addition of lignin at 0.005–0.02%, reaching 43.2%, 39.4%, and 38.9%, respectively, compared to the polymer blend of PLA and PBAT without DCP, (weight loss of 8.7% over the same period). Notably, when DCP was added at 0.03%, the weight loss was 14.0%.

Based on Figure 14, the polymer began to show physical changes after 30 days of soil burial. The lignin-containing polymers started to crack and their color changed. In contrast, the PLA and PBAT polymers without lignin showed only minor changes, with slight holes or discoloration at 60 days. The slight weight change may have been due to changes in characteristics such as brittleness and opacity. Although lignin itself is relatively resistant to biodegradation due to its complex aromatic structure, small amounts of lignin can indirectly enhance the biodegradation of polymer blends. Lignin particles may increase surface roughness and introduce microstructural heterogeneity in the polymer matrix, facilitating microbial colonization and accelerating degradation of the surrounding biodegradable polymers such as PLA and PBAT. In addition, lignin particles can act as localized stress concentration sites, promoting microcrack formation and increasing the accessible surface area for microbial attack. Although lignin degradation may produce aromatic compounds during microbial or fungal breakdown, the lignin content used in the current study was very low (0.005–0.02 wt.%); therefore, the potential ecological impact could be expected to be minimal. Consistent with these mechanisms, the films exhibited surface cracking, discoloration, and increased brittleness after soil burial, indicating progressive structural degradation of the polymer matrix [4].

### 3.8. Life Cycle Assessment of PLA/PBAT Polymer

**Figure 15:**
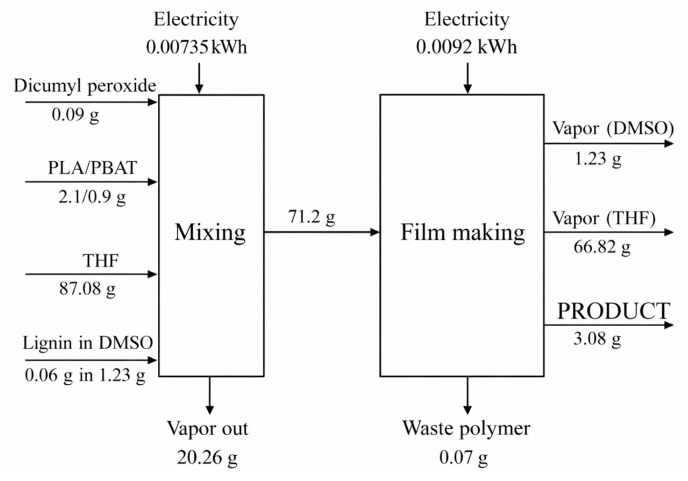
Process of lignin PLA/PBAT production, mass balance, and energy consumption.

The process of polymer film production was assessed based on the initial materials used (Figure 15), the forming process, and the final steps to obtain the film product. The environmental impact of PLA was obtained from the database. The PBAT was evaluated from the esterification synthesis of monomers from 1,4-butanediol (BDO), adipic acid (AA), and terephthalic acid (TA) as displayed in Equations (3) and (4), respectively. In these reactions, producing 1 kg of PBAT (Equation (5)) required 0.3333 kg of AA, 0.4110 kg of BDO, and 0.3788 kg of TA, with 0.0411 kg of water being produced.

AA + BDO → A monomer + water(3)

TA + BDO → B monomer + water(4)

A monomer + B monomer → PBAT + water(5)

Figure 15 presents the process diagram of this lignin PLA/PBAT film production. This two-step process started with mixing all the ingredients: dicumyl peroxide, PLA/PBAT, THF (solvent), and a solution of lignin in DMSO, based on [17], with an electricity consumption of about 0.0418 kWh/kg of film product [17].

**Figure 16:**
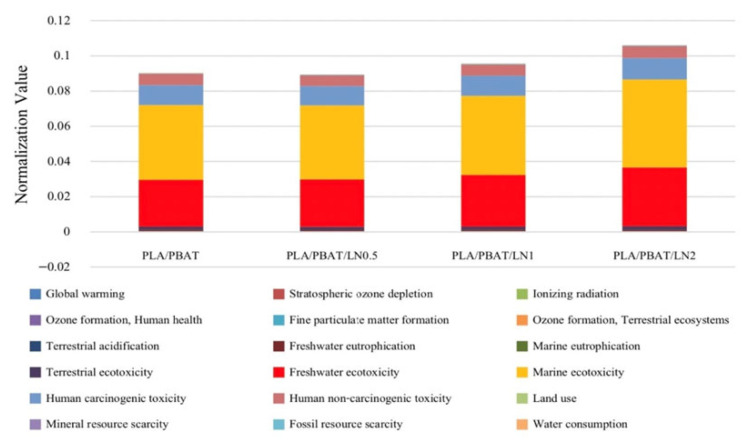
Impact of PLA/PBAT polymer blends with different amounts of lignin assessed using the ReCiPe Midpoint (H) model.

The environmental performance of the PLA/PBAT polymer film and lignin was based on a comparison of the results for each lignin ratio. As shown in Figure 16, the 0.005% lignin level produced the lowest overall impact, whereas the 0.02% lignin level produced the highest impact. Therefore, the minimum lignin amount of 0.05% was chosen. Based on the analysis of the results, the main implications in descending order were ecotoxicity to marine and freshwater ecosystems, carcinogenic toxicity to humans, and non-carcinogenic toxicity to humans. The evaluation of the impact on marine and freshwater ecosystems of the PLA and PBAT polymer films based on the different lignin ratios indicated quite similar values (0%, 0.043827731 and 0.032663942 kg 1,4-DCB, respectively; 0.005%, 0.043431962 and 0.033030394 kg 1,4-DCB, respectively; and 0.02%, 0.051502057 and 0.040996882 kg 1,4-DCB, respectively).

**Figure 17:**
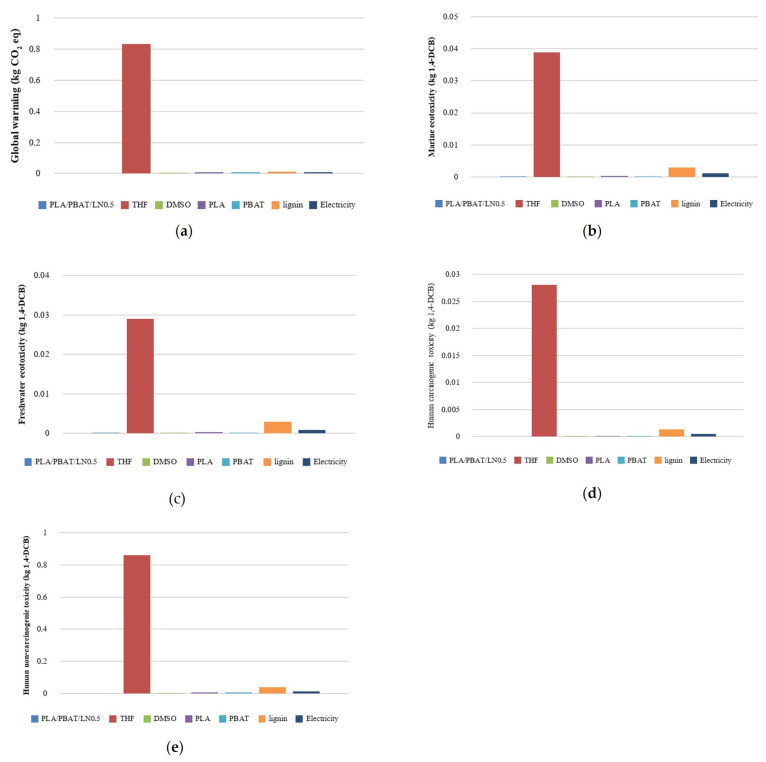
Environmental impacts of various aspects of PLA/PBAT/DCP3/LN^0.5^ polymer using the ReCiPe Midpoint (H) model: (**a**) global warming; (**b**) marine ecotoxicity; (**c**) freshwater ecotoxicity; (**d**) human carcinogenic toxicity; (**e**) human non-carcinogenic toxicity.

In conclusion, considering the various impacts, it was not conclusive which aspect had the highest impact. Therefore, it was necessary to focus the analysis on the most affected aspect, which was determined from the normalization value graph (Figure 17), which indicated the main factor for polymer film molding was the solvents, specifically THF.

The analysis on global warming in the polymer film ratios of PLA, PBAT, with lignin at 0.005% (PLA/PBAT/DCP3/LN^0.5^), as shown in Figure 17a, revealed that the main impact came from the THF solvent (0.834056915), followed by lignin (0.012350825), PLA (0.00857958), electricity (0.008502596), PBAT (0.008388915), and finally, DMSO (0.000770268 kg 1,4-DCB).

Regarding the environmental impact on marine ecotoxicity, as shown in Figure 17b, the main implications also came from THF (0.038963844), followed by lignin (0.002923299), electricity (0.001020892), PLA (0.000352827), PBAT (0.00010127), DMSO (3.68539 × 10^−5^), and finally, polymer film (3.29762 × 10^−5^ kg 1,4-DCB).

Regarding the environmental impact on freshwater ecotoxicity, as shown in Figure 17c, the main implications again came from THF (0.02900303), followed by lignin (0.002840297), electricity (0.000808265), PLA (0.000284419), PBAT (6.52721 × 10^−5^), DMSO (2.80582 × 10^−5^), and finally, polymer film (1.05266 × 10^−6^ kg 1,4-DCB). A similar pattern was observed for the environmental impact on both carcinogenic and non-carcinogenic toxicity to humans (Figure 17d,e), with the descending order of impacts being THF, lignin, electricity, PLA, PBAT, and DMSO.

THF was used as the solvent for the PLA, PBAT, and DCP mixtures. In addition, THF is common in many polymer processes because of its versatility, high volatility (boiling point of 66 °C), and strong solvency [29]; however, it has the greatest negative environmental impact, as shown in Figure 17. Alternative chemicals, including 2-methyltetrahydrofuran (2-MeTHF) and 2,5-dimethyltetrahydrofuran (DMeTHF), have been proposed [29]. In the current study, lignin had the second-largest environmental impact; however, it improved the biodegradability of the composites (see weight loss in Figure 13 and physical degradation in Figure 14). The development of new lignin extraction techniques is a necessary challenge to reduce the overall environmental impacts of lignin.

## 4. Conclusions

This study developed biodegradable polymer films composed of poly(lactic acid) (PLA) and poly(butylene adipate-co-terephthalate) (PBAT) in a 70:30 ratio, using dicumyl peroxide (DCP) as a compatibilizer and lignin as a functional additive. The addition of DCP improved the interfacial adhesion between PLA and PBAT in the samples; however, excessive DCP (above 0.03%) slightly reduced mechanical performance due to the increased crosslinking density. Lignin incorporation enhanced thermal stability and biodegradability, with the optimum content of 0.005% yielding the best balance among tensile strength, elongation at break, and elastic modulus. Potential applications of the synthesized material (PLA/PBAT/DCP3/LN^0.5^) include biodegradable packaging materials, easy-tear plastic films, protective food packaging, and UV-shielding packaging materials. Life cycle assessment indicated that solvent use caused the main environmental impacts, primarily marine and freshwater ecotoxicity, while the global warming potential remained minimal. Overall, lignin-modified PLA/PBAT/DCP/LN^0.5^ films showed promise as sustainable alternatives for UV absorbers and biodegradable packaging. Further research should focus on substituting THF with alternative chemicals, such as 2-MeTHF and DMeTHF, to develop safe and clean processes and to reduce the environmental impact of lignin through the innovative development of the extraction process.

## Figures and Tables

**Table 1:** Sample codes and dicumyl peroxide (DCP) concentrations at PLA-to-PBAT ratio of 70:30.

Sample	DCP (wt.%)
PLA/PBAT/DCP0	0
PLA/PBAT/DCP1	0.01
PLA/PBAT/DCP2	0.02
PLA/PBAT/DCP3	0.03

**Table 2:** Sample codes and lignin (LN) concentration at PLA-to-PBAT ratio of 70: 30 with 0.03% DCP (DCP3).

Sample	Lignin (wt.%)
PLA/PBAT/DCP3	0
PLA/PBAT/DCP3/LN^0.5^	0.005
PLA/PBAT/DCP3/LN^1^	0.01
PLA/PBAT/DCP3/LN^2^	0.02

**Table 3:** Thermal stability of PLA and PBAT polymer films with varying DCP concentrations (0, 0.01, 0.02, and 0.03%) using TGA.

Sample	T_5%_ (°C)	T_50%_ (°C)	T_d_ (°C)	Char Residual (%)
PLA/PBAT	276.8	342.1	420.7	-
PLA/PBAT/DCP1	286.7	343.3	421.9	1.5
PLA/PBAT/DCP2	293.4	350.1	423.5	2.1
PLA/PBAT/DCP3	172.2	346.1	423.5	-

**Table 4:** Thermal stability of PLA/PBAT polymer films with 0.03% DCP and lignin at different concentrations (0.005–0.02%).

Sample	T_5%_ (°C)	T_50%_ (°C)	T_d_ (°C)	Char Residual (%)
PLA/PBAT/DCP3/LN^0.5^	286.2	344.4	427.5	2.2
PLA/PBAT/DCP3/LN^1^	278.4	346.3	431.6	3.8
PLA/PBAT/DCP3/LN^2^	166.2	339.4	423.5	2.4

**Table 5:** UV-vis absorption, UV absorption edge wavelength, and band gap energy (E_g_) of polymer films with different lignin concentrations.

Sample	Absorbance at 600 nm	Absorption Edge Threshold (nm)	Band Gap Energy; E_g_ (eV)
PLA/PBAT	1.3 ± 0.06	302.3	4.1 ± 0.01
PLA/PBAT/DCP3	1.4 ± 0.09	316.2	3.9 ± 0.03
PLA/PBAT/DCP3/LN^0.5^	1.3 ± 0.00	310.2	4.0 ± 0.06
PLA/PBAT/DCP3/LN^1^	1.5 ± 0.01	315.5	3.9 ± 0.05
PLA/PBAT/DCP3/LN^2^	1.9 ± 0.01	312.8	3.9 ± 0.04

## Data Availability

The original contributions presented in this study are included in the article. Further inquiries can be directed to the corresponding author.
